# Olfactory Selection Preferences of *Pagiophloeus tsushimanus* (Coleoptera: Curculionidae) Adults Toward Lauraceae Plants

**DOI:** 10.3390/life14111517

**Published:** 2024-11-20

**Authors:** Cong Chen, Zhaoyan Lin, Jingyi Du, Jingyi Huang, Chunmei Ling, Jianfeng Chen

**Affiliations:** College of Life Sciences, Zhaoqing University, Zhaoqing 526061, China; 202224081357@stu.zqu.edu.cn (Z.L.); 202124081360@stu.zqu.edu.cn (J.D.); 202224081104@stu.zqu.edu.cn (J.H.); 202224081329@stu.zqu.edu.cn (C.L.); 202224081160@stu.zqu.edu.cn (J.C.)

**Keywords:** *Pagiophloeus tsushimanus*, *Cinnamomum camphora*, *Cinnamomum chekiangensis*, *Phoebe chekiangensis*, host transfer, wood-boring pest, electroantennography

## Abstract

*Cinnamomum camphora* is a broad-spectrum insect-repelling tree species because of its high content of terpenoids. However, it is curious that *Pagiophloeus tsushimanus*, a recently recorded wood-boring pest, has widely infested *C. camphora* plantations across various administrative districts in Shanghai. The larvae, being concealed within the trunk of *C. camphora* trees, exhibit characteristics such as hidden activity, strong destructiveness, and being difficult to control. While the primary host plant for *P. tsushimanus* is *C. camphora*, preliminary observations have shown that the pest can also complete its life cycle on *Cinnamomum chekiangensis* and *Phoebe chekiangensis*. To explore the host selection preference of this beetle, the present study aimed to investigate the olfactory selection behavior of *P. tsushimanus* adults towards *C. camphora*, *C. chekiangensis*, and *P. chekiangensis*. Results from choice and no-choice tests indicated that both male and female adults exhibited a feeding preference for *C. camphora* twigs, with females showing a preference for laying eggs on camphor tree twigs as well. Volatile compounds unique in camphor trees were significantly higher in relative content than those on the other two plants that were detected. The Y-shaped olfactometer experiments and electroantennography measurements results showed that male and female adults of *P. tsushimanus* had a positive chemotaxis towards volatiles released by *C. camphora* and a negative chemotaxis towards volatiles released by *C. chekiangensis* or *P. chekiangensis*. Overall, the findings suggest that both male and female adults have a selective preference for volatiles released by the camphor tree, and this provides a theoretical basis for monitoring and controlling the occurrence of this weevil pest.

## 1. Introduction

The camphor tree, *Cinnamomum camphora* (Laurales: Lauraceae) is one of the important ornamental and greening tree species and is extensively planted in southern China [1]. Due to its rich content of chemical defense substances, such as camphor, *C. camphora* is generally resistant to various pest insects [1,2,3]. However, the practice of cultivating large areas of monoculture *C. camphora* plantations has led to a uniformity in forest structure, which in turn has weakened the trees’ resistance to pests. An increase in pest species damaging camphor tree forests has been reported, including *Pagiophloeus tsushimanus* (Coleoptera: Curculionidae), *Orthaga achatina* (Lepidoptera: Pyralidae), and *Orthaga olivacea* (Lepidoptera: Pyralidae) [1,2,3,4,5,6]. *Pagiophloeus tsushimanus* was first reported in China, and this bores into and damages camphor trees [4,7,8]. Both adults and larvae can damage camphor trees. After emerging, adults climb towards the canopy to feed on the tender outer bark of twigs, and then larvae feed on the phloem and cambium, producing large amounts of frass [4]. The collective damage from both adults and larvae significantly reduces the economic and aesthetic value of *C. camphora*, and high pest densities can weaken trees and even cause them to die [4,9]. *Cinnamomum camphora* forests across multiple administrative districts in Shanghai have experienced significant infestation by this pest, exhibiting a spreading pattern that poses a considerable threat to camphor tree resources in adjacent regions [4,10].

Research on *P. tsushimanus* has thus far been limited, with studies primarily concentrating on pest identification, damage characteristics, life cycle, adult mating and oviposition behaviors, developmental stages, and genes function [4,7,9,10,11,12,13]. However, there have been no studies reported on the pest’s preference for different plants within the Lauraceae family, leaving an assessment of the risk of host shifting unexplored. Nutritional components such as proteins, sugars, and amino acids differ among plants, and these variations can affect the growth, survival, and reproduction of insects, thereby influencing their adaptability to different plants [13,14]. Phytophagous insects and their host plants undergo continuous natural selection and mutual adaptation through long-term coevolution, leading to differences in insect preferences for various plants [15]. The choice of suitable host plants directly impacts the survival and development of the insects, the growth performance of their offspring, and even the dynamics of the entire population [16,17,18]. In scenarios where the preferred host is absent, female insects may be compelled to lay eggs on non-host plants. If the larvae successfully adapt to some of these non-hosts, the emerging females may continue to choose these new hosts for oviposition, leading to host shifts [19]. Field surveys have found that this weevil pest feeds exclusively on the camphor tree, but indoor rearing experiments have shown that it can also complete its life cycle on other Lauraceae plants such as *Cinnamomum chekiangensis* and *Phoebe chekiangensis* [10]. This indicates that these species of plants could serve as potential alternative hosts and pose a risk of host shifting [9]. Three species of Lauraceae plants (*C. camphora*, *C. chekiangensis*, and *P. chekiangensis*) share the same distribution areas in Southeast China and are important urban greening trees [3,9,20]. Therefore, this study examines the feeding, oviposition, and olfactory behavioral responses of adults of *P. tsushimanus* toward these three species of plants, aiming to assess the potential risk of host shifting and to provide a reference for monitoring pest damage.

## 2. Materials and Methods

### 2.1. Main Instruments and Equipment

Instruments used include an atmospheric sampler (QC-1B, Beijing Labor Protection Science Research Institute, Beijing, China) and a Y-shaped olfactometer (custom-made by Nanjing Zebra Experimental Equipment Co., Ltd., Nanijing, China) The Y-shaped olfactometer is made of glass composed of an inner diameter (4 cm), two side arms each 20 cm long at a 60-degree angle to each other, and a main arm (length 40 cm). The ends of the side arms are connected to the odor source bottles through Teflon tubing. Air is first drawn through an activated carbon filter to purify it, followed by a distillation water filter to increase humidity, using the atmospheric sampler. An equal airflow rate (300 mL/min) passes through either the odor source or control and then converges in the main arm tube from the side arms, entering the atmospheric sampler through the main arm’s outlet.

### 2.2. Test Insects

Larvae and pupae of *P. tsushimanus* were collected from 15-year-old camphor trees in southern China. They were brought back to the laboratory and reared individually in 6-well plates (2.5 cm diameter per well) until the adults’ emergence. Larvae and pupae were placed in appropriate conditions (26 ± 1 °C, 60 ± 5% relative humidity, 0: 24 h light photoperiod) and larvae were provided with artificial diet feed containing *C. camphora* twigs debris. Adults were placed in plastic containers (60 cm long, 30 cm wide, 20 cm high) under appropriate conditions (26 ± 1 °C, 60 ± 5% relative humidity, 14:10 h light photoperiod) and reared in fresh 2-year-old tender *C. camphora* twigs, which were replaced every 3 days. Adults that emerged on the same day were selected for subsequent experiments.

### 2.3. Selectivity Tests of P. tsushimanus Adults on Three Species of Plants

#### 2.3.1. Choice Test

Unmated healthy male adults (36) and female adults (36) were selected for testing. Before testing, these insects were starved for 24 h. The plants (*C. camphora*, *C. chekiangensis*, and *P. chekiangensis*) chosen for the test were used to determine adult feeding and oviposition preferences. Each rearing box (6 cm height × 12 cm wide × 18 cm long), containing 6 male and 6 female adults, was simultaneously furnished with 10 cm-long twigs of the three species of plants for the insects to choose from, with twigs replaced weekly. There were three replicates, and two rearing boxes belonged to one repetition. Daily observations and recordings were made of the feeding area and egg-laying quantity of the three species of plants for three months. The area fed upon was calculated using a method involving grid paper coated with sulfuric acid, tallying the marked squares [21].

#### 2.3.2. No-Choice Test

The status of the test insects and the pre-experiment handling methods were the same as in 2.3.1. The adults’ feeding areas and oviposition quantities were measured on single species of Lauraceae plants. Each rearing box (same dimensions as Section 2.3.1, containing 6 male and 6 female adults) was separately furnished with twigs of three species of plants (same dimensions as Section 2.3.1) for the insects to feed on, with twigs replaced weekly. Three replicates were set for each species of plant, with each replicate containing two rearing boxes. Daily observations and recordings were made of the feeding area and egg-laying quantity of the three species of plants for three months.

### 2.4. Behavioral Response Measurements of P. tsushimanus Adults to Three Species of Plants

#### 2.4.1. Behavioral Responses of Adults to Three Species of Plants and Air Control

The status of the test insects and the pre-experiment handling methods were the same as in Section 2.3.1. Approximately 10 cm-long, 2-year-old twigs of three species of plants, each with five twigs, were placed separately in one of the odor source bottles of the Y-shaped olfactometer. In contrast, the other bottle was left empty as an air control. Before the test, acetone was used to clean the components of the Y-tube olfactometer, and the components were then thoroughly rinsed in distilled water and dried. Two branch arms of the Y-tube olfactometer needed be reversed after each test. During the experiment, a single adult (female or male) was placed at the entrance of the main arm. Before the test, the atmospheric sampler was run for 30 s. If an adult moved beyond the halfway mark of a selected arm and remained active for ≥3 min near the odor source area, it was recorded as having chosen the odor source connected to that arm. If the insect did not enter the marked area within 5 min, it was recorded as a no-choice (not used for calculation). Three repetitions were set up, and each repetition contained five new twigs of one species of Lauraceae plants and was tested with adults from two rearing boxes, each box contained 6 female and 6 male adults. Each insect was only used for testing once. The device was placed maintained at condition (2000 lx, 26 ± 1 °C, uniform light).

The formula for calculating the selection response rates is

$$SRR=\frac{ST}{ST+CT}$$

where *SRR* is selection response rates; *ST* is the number of adults that select to enter the odor source tube; and *CT* is the number of adults in the control tube [22].

The formula for calculating the selection coefficient is

$$SC=\frac{ST-CT}{ST+CT}$$

where *SC* is the selection coefficient; *ST* is the number of adults that select to enter the odor source tube; and *CT* is the number of adults in the control tube [22].

Positive values in the selection coefficient calculations indicate positive chemotaxis, while negative values indicate negative chemotaxis.

#### 2.4.2. Behavioral Response of Adults Among Three Species of Plants

The condition of the test insects and the pre-experiment handling methods were the same as in Section 2.3.1. For each test, five twigs that were approximately 10 cm long and 2 years old of one of three species of plants were placed in one of the odor source bottles of the Y-shaped olfactometer, while twigs of another plant were placed in the other bottle. Three comparative groups were established: *C. camphora* vs. *C. chekiangensis*, *C. camphora* vs. *P. chekiangensis*, and *C. chekiangensis* vs. *P. chekiangensis*. Three repetitions were set up, and each repetition contained five new twigs of one species of Lauraceae plants and five new twigs of the other species. Each repetition was tested with 12 adults (6 female and 6 male adults). Each insect was only used for testing once. The testing and calculation methods followed those described in Section 2.4.1.

### 2.5. Extraction and Identification of Volatile Compounds Released by Lauraceae Plants

The dynamic headspace adsorption method was used to collect and extract volatile compounds from the two-year-old tender twigs of three species of plants [23]. This experiment was set up with six replicates, each containing 20 branches. Before use, the Porapak Q and traps were cleaned with Soxhlet-extracted dichloromethane. The airflow was filtered by charcoal before entering the device, which entered the 500 mL conical flask (containing tender twigs) or flask (empty control) at a flow rate (350 ml/min) provided by an atmospheric air collection pump (Beijing Municipal Institute of Labour Protection, Beijing, China). The traps near the air outlet consisted of glass tubes filled with Porapak Q adsorbent (80/100 mesh; 200mg; Sigma-Aldrich, St. Louis, MO, USA). The twig-generated volatiles were collected for 24 h. A total of 1 mL Dichloromethane was used to elute the trapped volatiles from each adsorption tube. Eluent was loaded into a vial for chromatography samples. The extract was concentrated using the nitrogen-blowing method to increase the concentration of volatile compounds. Each vial was sealed before being used for each experiment. 

Coupled gas chromatography-mass spectrometry (GC-MS) (Thermo Electron, USA) was used to identify volatile extracts derived from *C. camphora*, with the following parameters: (1) The injector and detector temperature was 250 °C using splitless injection. (2) High-purity nitrogen was used for carrier gas (flow rate of 2 mL/min). (3) The program of oven started from 40 °C, then was ramped up to 80 °C at a rate of 10 °C per min, to 100 °C at a rate of 5 °C per min, to 105 °C at a rate of 1 °C per min, to 120 °C at a rate of 5 °C per min, and finally to 250 °C (3 min hold time) at a rate of 10 °C per min. EI mode (70 eV) was used for mass spectra. The temperatures of both the transfer line and ion source were 250 °C. The scanning ranged from 30 to 350 m/z. All volatile compounds were identified by matching mass and database spectra, and then matching of key compounds was confirmed by the retention time, mass spectrum, and authentic standards.

### 2.6. Behavioral Response Measurements of P. tsushimanus Adults to Four Volatile Components Released by Lauraceae Plants

The olfactory behavioral responses of *P. tsushimanus* female and male adults to a single component of four compounds were measured by a Y-shaped olfactometer. The test insects and the pre-experiment handling methods were the same as in Section 2.3.1. The testing method of the Y-shaped olfactometer and calculation methods followed those described in Section 2.4.1. Four representative volatile compounds were selected and prepared into solutions with concentrations of 10 μg/μL using liquid paraffin, such as β-caryophyllene (released by three species of plants, but relatively high in camphor trees), linalool (released by *C. camphora* and *C. chekiangensis*, but not released by *P. chekiangensis*), trans-nerolidol (only released by camphor trees *C. camphora*, but not released by the two other trees *C. chekiangensis* and *P. chekiangensis*), and α-phellandrene (only released by *C. camphora*). A total of 10 μL of a given volatile compound (one of four compound solutions with concentrations of 10 μg/μL) was applied to a strip placed separately in one of the odor source bottles of the Y-shaped olfactometer, while 10 μL of liquid paraffin (control treatment) was applied in the other bottle. 

### 2.7. Electroantennography (EAG) Responses Measurements of P. tsushimanus Adults to Four Volatile Components Released by Lauraceae Plants

In the laboratory, EAG was used to measure the electrophysiological responses of *P. tsushimanus* to single component of *C. camphora* trees volatiles in order to screen for components with antennal electrophysiological activity. Based on the comparative analysis in 2.5, combined with previous research reports on volatile compounds in the camphor tree, linalool, trans-nerolidol, α-phellandrene, and β-caryophyllene are the main differential volatile components of three species of plants [1,24]. Therefore, these four volatile compounds of camphor trees were selected as the objects for EAG determination. 

Four compounds (linalool, trans-nerolidol, α-phellandrene, and β-caryophyllene) were prepared into solutions (0.001, 0.01, 0.1, 1, and 10 μg/μL) using liquid paraffin. EAG responses of antennae flagellomeres of adults to all treatments were tested. The EAG testing method refers to previously reported methods [25,26]. DC mode with combi probe (Ockenfels syntech gmbh, prg-3) (Syntech Corporation, Büchenbach, Germany) was used for electric continuity between antennae and the recording equipment (EAG 2.0 program, Syntech Laboratories, Hilversum, the Netherlands). The air was filtered by being charcoal-filtered, humidified, and blown, passing through a stainless-steel delivery pipe (1 cm inner diameter) onto the antenna (seventh to ninth segments) at a flow rate of 500 mL/min.

A new Pasteur pipette was connected to the stimulus source tube with a strip containing 10 μL of volatile compound. The pedal was gently pressed and recording was started. Each stimulation was set at a flow rate of 60 mL/s, lasted for 0.2 s, and the interval between the two stimuli should have been 60 s [26]. A total of 10 μL of liquid paraffin (control treatment) was tested, respectively, in-between tests. Each treatment was tested on antennae of male adults (*n* = 20) and antennae of female adults (*n* = 20). Each antenna was tested 3 times. 

### 2.8. Data Analysis

EAG reaction value analysis is based on the methods reported in previous studies [26,27,28]. The detailed operations are as follows: the maximum amplitude of negative polarity deflection (-mV) was used to measure the EAG response. The resulting EAG values were corrected by reducing the EAG amplitude to a standard stimulus to compensate for the decrease in antennal reactivity during the experiment.

ANOVA with post-hoc Tukey HSD test were used for statistical analysis of feeding area, oviposition quantity, and selection coefficient. Chi-square test were used to compare the selection response rates of male and female adults to different odor sources; Student’s *t*-tests were used to compare the selection coefficients between male and female adults to different odor sources. All statistical analyses were conducted in SPSS 20.0 software.

## 3. Results

### 3.1. Feeding Preferences of P. tsushimanus Adults Toward Three Species of Plants

**Figure 1:**
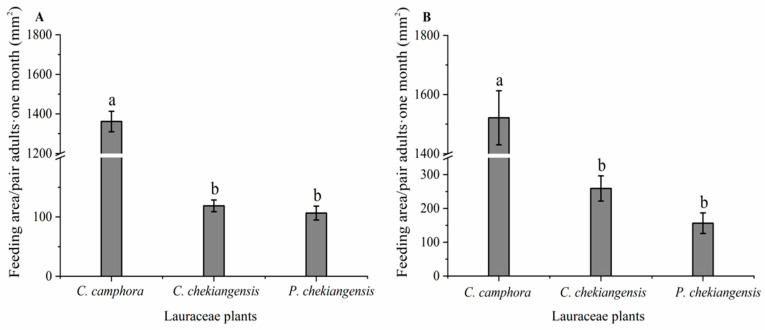
Feeding area of female and male adults on three species of plants under choice (**A**) and no-choice conditions (**B**). Different small letters indicate that there is significant difference under the condition of *p* < 0.05 by ANOVA with post-hoc Tukey HSD Test.

As indicated in Figure 1, under both choice (*F* = 534.791; *p* < 0.01) and no-choice (*F* = 162.260; *p* < 0.01) conditions, significant differences were observed in the feeding areas on twigs of three species of plants by female and male adults of *P. tsushimanus*. Under choice conditions, adults significantly preferred feeding on *C. camphora* compared to *C. chekiangensis* and *P. chekiangensis*, with no significant difference in the feeding area between *C. chekiangensis* and *P. chekiangensis*. Similarly, under no-choice conditions, the feeding area of *C. camphora* was significantly larger compared to both *C. chekiangensis* and *P. chekiangensis*, with no significant differences between *C. chekiangensis* and *P. chekiangensis*.

### 3.2. Oviposition Preferences of P. tsushimanus Female Adults Toward Three Species of Plants

**Figure 2:**
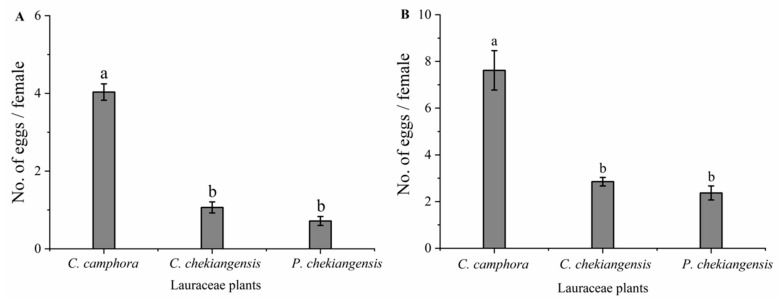
Oviposition of female adults on three species of plants under choice (**A**) and no-choice conditions (**B**). Different small letters indicate that there is significant difference under the condition of *p* < 0.05 by ANOVA with post-hoc Tukey HSD Test.

Under both choice (*F* = 129.096; *p* < 0.01) and no-choice (*F* = 35.835; *p* < 0.01) conditions, significant differences were observed in the number of eggs laid per female per month on twigs of three species of plants of *P. tsushimanus* female adults (Figure 2). Under both conditions, the egg-laying rate on *C. camphora* twigs was significantly higher than on the other two plants, while no significant differences were found between *C. chekiangensis* and *P. chekiangensis* (Figure 2). Under non-selective conditions, the oviposition rate on *C. chekiangensis* was significantly higher than under selective conditions; however, the oviposition rates on *C. chekiangensis* and *P. chekiangensis* did not differ significantly between selective and non-selective conditions.

### 3.3. Behavioral Responses of P. tsushimanus Adults to Three Species of Lauraceae Plants

#### 3.3.1. Behavioral Responses of Adults to One of Three species of Plants Versus Air Control

**Figure 3:**
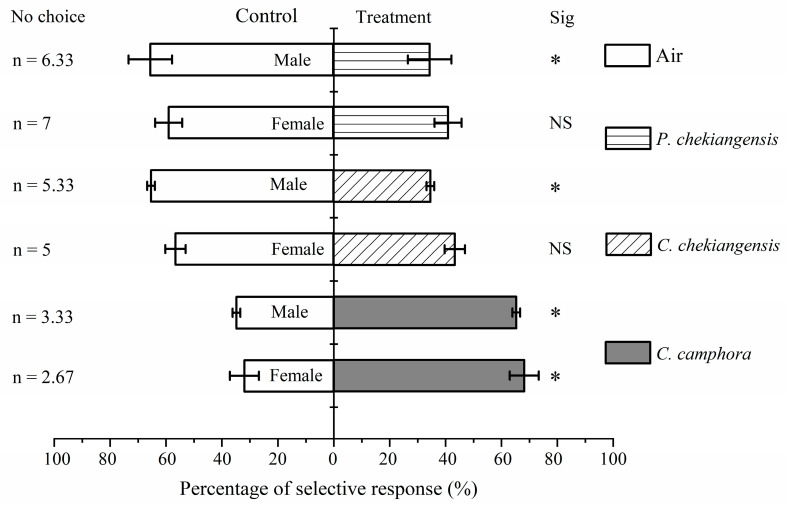
Olfactory response of female and male adults between one of three species of plants and the air control. Asterisk indicates that the selection response rate between the treatment group and the control group has significant difference under the condition of *p* < 0.05 using Chi-square test. NS refers to no significant difference.

Figure 3 shows that both female (*χ*^2^ = 5.974, *p* < 0.01) and male (*χ*^2^ = 4.010, *p* < 0.05) adults exhibited a significantly higher response rate towards *C. camphora* compared to the control. Females did not show a significant preference for either *C. chekiangensis* (*χ*^2^ = 0.724, *p* > 0.05) or *P. chekiangensis* (*χ*^2^ = 1.290, *P* > 0.05) compared to the control, while males demonstrated a significantly lower preference rate towards *C. chekiangensis* (*χ*^2^ = 4.010, *p* < 0.05) and *P. chekiangensis* (*χ*^2^ = 4.010, *p* < 0.05) compared to the control. There were no significant differences in the response rates towards *C. camphora* (*χ*^2^ = 0.106, *P* > 0.05), *C. chekiangensis* (*χ*^2^ = 0.403, *p* > 0.05), or *P. chekiangensis* (*χ^2^* = 0.655, *p* > 0.05) between males and females.

Table 1 shows that both female (selection coefficient = 0.363) and male (selection coefficient = 0.306) adults exhibited a positive chemotactic response towards *C. camphora*, indicating an attraction. Both females (selection coefficient = −0.131) and males (selection coefficient = −0.306) displayed a negative chemotactic response towards *C. chekiangensis*, suggesting repellence, and, similarly, both showed negative responses towards *P. chekiangensis* (female selection coefficient = −0.178, male selection coefficient = −0.310). There were no significant differences between females and males in the selection coefficients towards *C. camphora* (*t* = 0.535, *p* > 0.05), *C. chekiangensis* (*t* = 2.251, *p* > 0.05), or *P. chekiangensis* (*t* = 0.717, *p* > 0.05).

#### 3.3.2. Behavioral Responses of Adults to Pairs of Three Species of Plants

**Figure 4:**
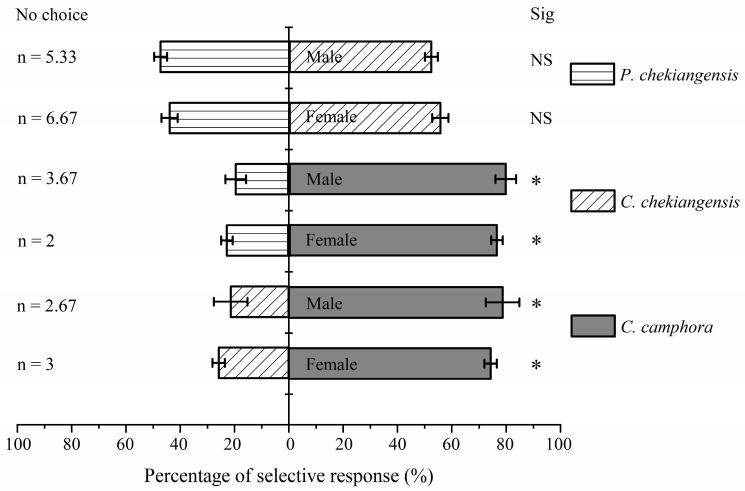
Olfactory response of female and male adults to pairs of three species of plants. Asterisk indicates that the selection response rate between the treatment group and the control group has significant difference under the condition of *p* < 0.05 using Chi-square test. NS refers to no significant difference.

Figure 4 shows that both female (*χ*^2^ = 11.227, *p* < 0.05) and male (*χ*^2^ = 17.120, *p* < 0.05) adults significantly preferred *C. camphora* over *C. chekiangensis*. Similarly, the preference rates for *C. camphora* over *P. chekiangensis* were also significantly higher for both female (*χ*^2^ = 14.583, *p* < 0.05) and male (*χ*^2^ = 18.484, *p* < 0.05) adults. However, there was no significant difference in the preference rates between *C. chekiangensis* and *P. chekiangensis* for both female (*χ*^2^ = 0.502, *p* > 0.05) and male (*χ*^2^ = 0.020, *p* > 0.05) adults.

Table 2 indicates that the positive chemotactic responses of both female (selection coefficient = 0.485) and male (selection coefficient = 0.573) adults were stronger towards the camphor tree compared to *C. chekiangensis*, and for the camphor tree over *P. chekiangensis* (female selection coefficient = 0.537, male selection coefficient = 0.602). The positive chemotactic responses were also stronger towards *C. chekiangensis* compared to *P. chekiangensis* (female selection coefficient = 0.114, male selection coefficient = 0.048). There were no significant differences between females and males in their selection coefficients for the three species of plants (*C. camphora* vs. *C. chekiangensis*: *t* = −0.665, *p* > 0.05; *C. camphora* vs. *P. chekiangensis*: *t* = −0.748, *p* > 0.05; *C. chekiangensis* vs. *P. chekiangensis*: *t* = 0.875, *p* > 0.05).

### 3.4. Analysis of Different Volatile Compounds Among Three Species of Plants

According to Table 3, a total of 19 volatile compounds were detected. Six volatile components, including trans-nerolidol, Eucalyptol, (E)-4-hexen-1-ol, α-phellandrene, germacratrien-1-ol, and β-selinene, were only detected in *C. camphora* trees, while four volatile components, including sabinene, d-limonene, camphene, and (−)-β-pinene, were not detected in camphor trees. Four candidate volatile compounds, namely β-caryophyllene (released by three species of plants), linalool (released by two species of *C. camphora* and *C. chekiangensis*), trans-nerolidol (only released by *C. camphora*), and α-phellandrene (also only released by *C. camphora*) have been focused on and used to determine the antennal potential response of adults. 

### 3.5. Behavioral Responses of P. tsushimanus Adults to Four Volatile Components Released by Lauraceae Plants

**Figure 5:**
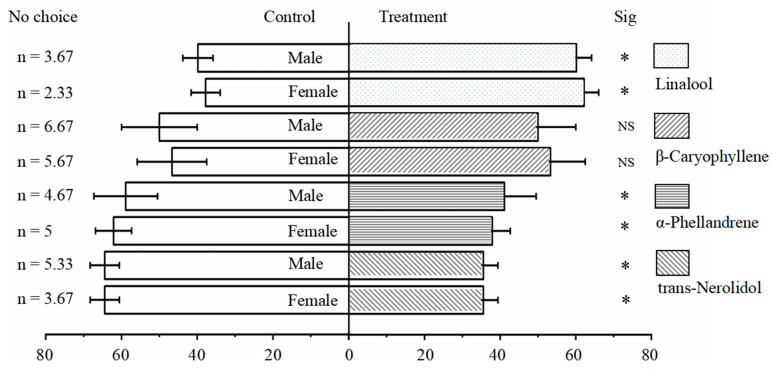
Olfactory response of female and male adults to linalool, β-caryophyllene, α-phellandrene, trans-nerolidol, and the air control. The asterisk indicates that the selection response rate between the treatment group and the control group has significant difference under the condition of *p* < 0.05 using Chi-square test. NS refers to no significant difference.

As shown in Figure 5, both female (*χ*^2^ = 6.237, *p* < 0.05) and male (*χ*^2^ = 4.163, *p* < 0.05) adults showed a significantly higher response rate towards linalool than that of the control, respectively. However, the selection response rate of male and female adults to α-phellandrene and trans-nerolidol was significantly lower than that of the control. There were no significant differences between females (*χ*^2^ = 1.147, *p* > 0.05) and males (*χ*^2^ = 1.029, *p* > 0.05) in the selection coefficients towards β-caryophyllene compared to the control.

According to Table 4, both females (selection coefficient = 0.244) and males (selection coefficient = 0.204) exhibited a positive chemotactic response towards linalool, indicating an attraction. Although male and female adults showed a positive tendency towards β-caryophyllene, the selection coefficient is very small, indicating weak attraction. Both females and males displayed a negative chemotactic response towards α-phellandrene and trans-nerolidol.

### 3.6. EAG Responses of P. tsushimanus Adults to Four Volatile Components Released by Lauraceae Plants

**Figure 6:**
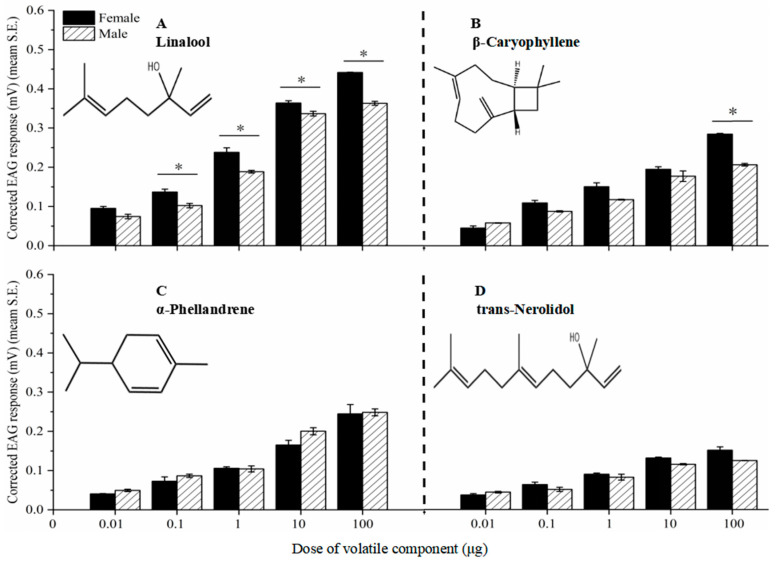
EAG responses of females and male antennae to four components. Component linalool (**A**), β-caryophyllene (**B**), α-phellandrene (**C**), and trans-nerolidol (**D**). The asterisk indicates significant differences between female and male adults (*p* < 0.05, Student’s *t*-tests).

The EAG response values of female and male adult antennae to four different concentrations of a single compound component increased with increasing concentration, and there were significant differences in the EAG response values between different concentrations (*p* < 0.05) (Figure 6).

There is a significant difference in the EAG response values between female and male adults for the same concentration of linalool (0.1, 1, 10, and 100 μg) (*t* = 3.759, *p* < 0.05; *t* = 4.055, *p* < 0.05; *t* = 3.313, *p* < 0.05; *t* = 15.48, *p* < 0.05) and β-caryophyllene (100 μg) (*t* = 20.291, *p* < 0.05) of the same compound. There was no significant difference in EAG response values between female and male adults for other compounds (Figure 6).

At a dose of 100 μg, the EAG response values of female adults for four single component compounds were as follows: linalool (0.4415 mV) > β-caryophyllene (0.2850 mV) > α-phellandrene (0.2447 mV) > trans-nerolidol (0.1515 mV). The EAG response values of male adults to four single components of compounds, from high to low, were as follows: linalool (0.3631 mV) > α-phellandrene (0.2483 mV) > β-caryophyllene (0.2068 mV) > trans-nerolidol (0.1249 mV) (Figure 6).

## 4. Discussion

Indoor experiments have found that beetles *P. tsushimanus* can complete their life cycle on three species of Lauraceae plants. Additionally, these three species of plants overlap in their distribution in the Southeast China region and are significant for urban greening in Shanghai and its surrounding areas [3]. Consequently, this study selected *C. chekiangensis* and *P. chekiangensis* as candidate plants to investigate the selection behavior of adults towards volatiles released by these Lauraceae plants, providing crucial insights for assessing the risk of host shifting in this pest.

*Cinnamomum camphora* has strong insect-repellent activities [1,2,3]. However, both female and male adults of *P. tsushimanus* showed a preference for feeding and females for ovipositing on camphor tree twigs under both choice and no-choice conditions. This preference could be attributed to the differences in the main secondary metabolites among these Lauraceae species [1,29,30], which influence adult feeding and oviposition behaviors [9]. Volatile chemical compounds play a pivotal role in guiding these behaviors [31], and non-volatile chemicals also significantly affect feeding and oviposition preferences, as evidenced by previous studies where larvae fed on semi-artificial diets made from twigs of these three species of plants. Larvae consuming diets containing camphor components had the shortest developmental periods, lowest mortality rates, and highest pupation rates and weight gain [9]. Female adults are likely to choose host plants that maximize the survival and growth potential of their offspring, a behavior driven by selective pressures aimed at maximizing offspring survival and population growth [32,33,34]. However, the choice of plants for feeding and oviposition by insects is also influenced by environmental factors such as altitude, slope orientation, temperature, humidity, and the physical characteristics of the plants, such as branch thickness. For example, the longhorn beetle *Aphrodisium sauteri* (Coleoptera:Cerambycidae) shows a preference for feeding on *Cyclobalanopsis myrsinaefolia* (Fagales: Fagaceae) and tends to oviposit on twigs with bark thicknesses of 1-2 mm and diameters of 15-25 mm [35]. Therefore, future studies on the oviposition habits of *P. tsushimanus* should consider a broader range of influencing factors.

Adults of *P. tsushimanus* can feed and oviposit on the twigs of *P. chekiangensis* and *C. chekiangensis*. In line with earlier research findings, larvae of the *P. tsushimanus* can complete their lifecycle when fed semi-artificial diets made from twigs of these two plants. This adaptability under suboptimal food conditions through physiological and biochemical adjustments suggests that *C. chekiangensis* and *P. chekiangensis* could serve as alternative hosts for adults, indicating a potential risk of host shifting [9,35]. Additionally, under pressures such as a scarcity of preferred host plants, *P. tsushimanus* might adapt to other species like *C. chekiangensis* and *P. chekiangensis* by modulating its growth, development, and detoxification metabolism [9]. Therefore, in field surveys monitoring the damage caused by this pest, there should also be an intensified effort to determine whether this weevil pest *P. tsushimanus* will harm other Lauraceae plants.

The selection of host plants by phytophagous insects is influenced by genetic factors, volatile compounds from host plants, and the growth and development performance of offspring [36,37]. Insects can genetically acquire the ability to recognize volatiles from host plants. For instance, Corbet (1985) proposed the early detection hypothesis of chemical residues, emphasizing that chemical residues from host plants are stored in the hemolymph of larvae or on the surface of pupae and are ultimately detected by adults after emergence [36,38]. Recent studies also suggest that the sensitivity to chemical cues exists in the central nervous system (CNS) of larvae and persists into adulthood [39]. However, there are opposing views suggesting that substantial reconstruction of the CNS during metamorphosis may prevent the transfer of chemical sensory memory [40,41]. The preference–performance hypothesis suggests that female adults of herbivorous insect species will select to lay eggs on host plants that have the best representation of their offspring [32,33]. 

However, some argue that the feeding and oviposition preferences of female adults may not necessarily correlate with the performance of their offspring [29,32]. Due to changes in the nutritional quality of plants over time, it becomes challenging for female adults to quickly determine the most suitable plants for the growth and development of their offspring, thus increasing the difficulty in recognizing changes in plant nutritional quality [1,42]. Volatile compounds from host plants play a crucial role in host recognition and oviposition site selection by adults, with the composition and concentration of these volatiles directly influencing adults’ choice behaviors [43,44]. Weevils exhibit selective preferences for volatiles from different plants; for example, *Sitophilus zeamais* (Coleoptera: Curculionidae) shows a stronger preference for *Triticum aestivum* (Poales: Poaceae) and *Arachis hypogaea* (Rosales: Leguminosae), *Fagopyrum esculentum* (Caryophyllales: Polygonaceae), and *Sorghum bicolor* (Cyperales: Poaceae) [45]. Significant electrophysiological response of female and male adults to four volatile compounds (linalool, trans-nerolidol, α-phellandrene, and β-caryophyllene) of the camphor tree was discovered in this study. Among them, female and male adults had a stronger electroantennogram response to linalool. In addition, both male and female adults showed significant behavioral responses to the volatile compound linalool, which has a higher relative content in the camphor tree compared to the other two plants (*C. chekiangensis* and *P. chekiangensis*). These results preliminarily indicate that male and female adults exhibit a positive chemotactic response to the volatile mix released by *C. camphora* but a negative chemotactic response to the volatile mix released by *C. chekiangensis* or *P. chekiangensis*. The olfactory perception mechanism of male and female adults towards the differential components of volatile compounds in Lauraceae plants will continue to be explored. At the same time, EAG and analytical chemistry methods will be used to isolate and identify key volatile components with host selection preferences from host plants, helping to develop attractants for controlling this pest and systematically analyzing the host selection preferences of *P. tsushimanus* adults.

The preference of insects for certain plants is associated with differences in the plants’ volatile compounds [30,31]. The relative content of four candidate compounds released by *C. camphora* is significantly higher than that of the other two plants. Two out of the four tested volatile components (α-phellandrene and trans-nerolidol) released from the preferred plant *C. camphora* (and not from the other two related plants) were avoided when tested singly at the tested concentration. The positive chemotactic response of *P. tsushimanus* adults to *C. camphora* volatiles suggests that key factors causing this phenomenon likely include significant differences in the volatile components or concentrations released by *C. camphora* compared to *C. chekiangensis* or *P. chekiangensis*. These results indicate that insects’ olfactory preference for plants is not only related to the type of compound but also to the concentration of the compound or the composition of mixed odors. Of course, it is also related to the excitatory effect of insects on compounds [46]. Identifying whether single or mixed components of these differences play a major role in adult olfactory choice requires further research. This study could help elucidate the mechanisms behind the preference of *P. tsushimanus* toward different plants and aid in the development of plant-based attractants for this pest. In addition, the behavior of insects is regulated by their genomes, so the selection preference of this pest for different plants can be analyzed from the perspective of the genome in the future [47].

## 5. Conclusions

*Pagiophloeus tsushimanus* shows olfactory selection preferences for *C. camphora* trees despite them being a broad-spectrum insect-repelling tree species. The volatile compounds released by *C. camphora* trees are the main factor triggering this olfactory selection preferences behavior. In the future, more key volatile compounds that determine the host preference behavior of *P. tsushimanus* will be further explored, and they will be applied to develop attractants and used for monitoring and controlling this weevil pest. The detoxification metabolic function of *P. tsushimanus* against non-volatile defense substances of Lauraceae plants will also be explored; it is of critical importance and significant meaning for the host preference adaptation of *P. tsushimanus*.

## Figures and Tables

**Table 1:** Selection coefficients of female and male adults between one of three species of plants and the air control.

Plant vs. Air	Selection Coefficients	
	Female Adults	Male Adults
*C. camphora* vs. air	0.363 ± 0.104	0.306 ± 0.028
*C. chekiangensis* vs. air	−0.131 ± 0.072	−0.306 ± 0.028
*P. chekiangensis* vs. air	−0.178 ± 0.097	−0.310 ± 0.156

Note: data represent values of mean ± standard deviation.

**Table 2:** Selection coefficients of female and male adults to pairs of three species of plants.

Plant vs. Another Plant	Selection Coefficients	
	Female Adults	Male Adults
*C. camphora* vs. *C. chekiangensis*	0.485 ± 0.046	0.573 ± 0.124
*C. camphora* vs. *P. chekiangensis*	0.537 ± 0.043	0.602 ± 0.076
*C. chekiangensis* vs. *P. chekiangensis*	0.114 ± 0.059	0.048 ± 0.048

Note: data represent values of mean ± standard deviation.

**Table 3:** Different volatile components among three species of plants.

Volatile Components	Relative Content/%			Cas Number
	*C. camphora*	*C. chekiangensis*	*P. chekiangensis*	
β-Caryophyllene	13.53 ± 0.19	11.22 ± 0.10	0.51 ± 0.12	87-44-5
Linalool	15.63 ± 0.20	2.72 ± 0.06	-	78-70-6
trans-Nerolidol	2.83 ± 0.15	-	-	40716-66-3
α-Phellandrene	1.51 ± 0.13	-	-	99-83-2
(+)-α-Pinene	0.40 ± 0.02	11.76 ± 0.06	15.99 ± 0.06	7785-70-8
α-Ocimene	1.94 ± 0.40	6.70 ± 0.21	0.53 ± 0.16	13877-91-3
Ocimene	19.34 ± 0.09	18.09 ± 0.10	17.93 ± 0.06	3779-61-1
(+)-Camphor	13.57 ± 0.18	3.23 ± 0.09	0.70 ± 0.09	464-49-3
Caryophyllene	4.02 ± 0.12	1.79 ± 0.31	-	1139-30-6
α-Humulene	2.48 ± 0.18	3.44 ± 0.18	-	6753-98-6
Eucalyptol	7.27 ± 0.06	-	-	470-82-6
(E)-4-Hexen-1-ol	1.59 ± 0.13	-	-	928-92-7
Germacratrien-1-ol	1.49 ± 0.26	-	-	81968-62-9
β-Selinene	1.64 ± 0.20	-	-	17066-67-0
Sabinene	-	5.63 ± 0.25	31.99 ± 0.12	3387-41-5
D-Limonene	-	9.27 ± 0.19	2.03 ± 0.09	5989-27-5
Camphene	-	3.86 ± 0.17	-	79-92-5
(−)-β-Pinene	-	-	18.41 ± 020	18172-67-3

Note: data represent values of mean ± standard deviation, and “-” means that the compound is not detected in plant volatiles.

**Table 4:** Selection coefficients of female and male adults to four volatile components.

Volatile Components vs. Air	Selection Coefficients	
	Female Adults	Male Adults
Linalool vs. Air	0.244 ± 0.770	0.204 ± 0.080
β-Caryophyllene vs. Air	0.067 ± 0.184	0.001 ± 0.200
α-Phellandrene vs. Air	−0.242 ± 0.095	−0.178 ± 0.168
trans-Nerolidol vs. Air	−0.289 ± 0.077	−0.289 ± 0.077

Note: data represent values of mean ± standard deviation.

## Data Availability

Data are contained within the article. The original contributions presented in the study are included in the article; further inquiries can be directed to the corresponding author.
